# Uniform pre-processing of bacterial single-cell RNA-seq

**DOI:** 10.64898/2025.12.04.692398

**Published:** 2026-01-16

**Authors:** Conrad Oakes, Vera Beilinson, Margaret J. McFall-Ngai, Lior Pachter

**Affiliations:** 1 Division of Biology and Biological Engineering, California Institute of Technology, Pasadena, CA, 91125, USA; 2 Division of Biosphere Sciences and Engineering, Carnegie Institution for Science, Pasadena, CA, 91125, USA; 3 Department of Computing and Mathematical Sciences, California Institute of Technology, Pasadena, CA, 91125, USA

## Abstract

Bacteria are highly heterogeneous, even under controlled conditions, making single-cell RNA sequencing (scRNA-seq) essential for studying microbial diversity and symbiosis. Since its first application in 2015, bacterial scRNA-seq has expanded, but different assays depend on distinct, custom, in-house pre-processing making it difficult to analyze data as part of a unified workflow. The kallisto-bustools suite of tools has enabled uniform pre-processing of eukaryotic scRNA-seq while also reducing time and resource demands for pre-processing, but is not optimized for bacterial scRNA-seq. We adapt kallisto-bustools to be suitable for reads generated from operons, as well as for a much shorter gene length distribution, and show that it can efficiently and accurately quantify bacterial scRNA-seq. Our work provides a scalable foundation for uniform pre-processing of microbial single-cell transcriptomics.

## Introduction

**Fig. 1.**
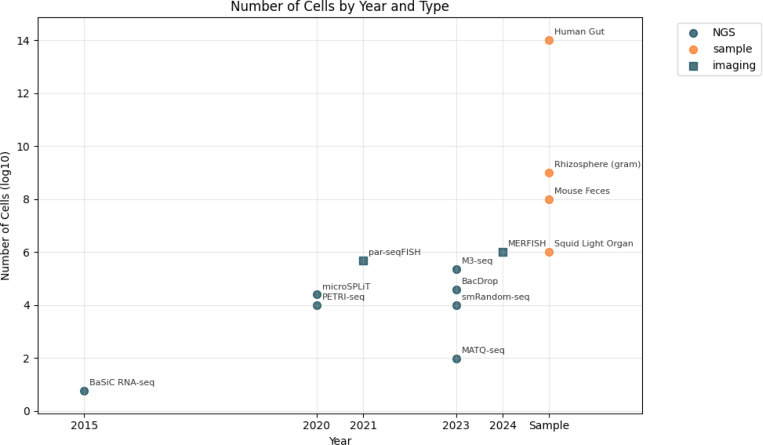
The number of cells analyzed was based on the values reported in each original method paper. For imaging-based approaches, cell numbers were determined by the practical limits of imaging throughput. Imaging-based methods are inherently targeted, requiring prior knowledge of the genes of interest to design appropriate probes. Biological samples (orange) do not have defined cell number; therefore, approximate values were used.

Single-cell RNA sequencing has transformed eukaryotic cell analysis by allowing an improved understanding of diversity at the individual-cell level. However, implementation of single-cell transcriptomics in bacteria has been limited due to a variety of biological and technical challenges, most significantly the lack of polyA tails. Recently, several methods have been developed that overcome these challenges, allowing untargeted sequencing of a bacterial RNA profile. With the growing recognition of the central role of host-bacteria interactions, bacterial single-cell transcriptomic datasets are poised to greatly increase in number (Figure 1).

The analysis of bacterial single-cell RNA-seq data can be challenging, starting with the pre-processing of reads for gene quantification. Bacterial genomes are frequently poorly annotated (1), leading to quantification errors due to unknown operons, and existing eukaryotic-optimized tools may not perform as expected due to incompatible parameter choices. In eukaryotes, genes are typically separated from each other by non-coding regions and are transcribed individually. In that context, a sequencing read is usually assigned to a single gene. Reads that map equally well to multiple genes are flagged as “multimapped” or “ambiguous” to avoid overcounting and artificially inflating expression levels. However, bacteria do not follow this one-gene-per-read rule and can frequently produce polycistronic transcripts from operons, thus sequencing reads can span multiple adjacent genes. Thus, current scRNA-seq pre-processing tools not only miscount genes but can also miss entire gene expression profiles.

We show that the kallisto-bustools (kb-python) suite of tools (2–4), which provide accurate and efficient pre-processing solutions for eukaryotic bulk and single-cell RNA-seq datasets via pseudoalignment, can be optimized for bacterial pre-processing and can recapitulate the results of other tools in a small fraction of the time. Moreover, we show that with kb, quantification can be performed for data from multiple different bacterial scRNA-seq technologies (specifically PETRI-seq (5), MATQ-seq (6), and BacDrop (7)), making possible uniform pre-processing of data generated by different laboratories for joint downstream analysis.

## Results

### PETRI-seq.

We analyzed data from (5) and found that quantification with kb-python proceeded at a rate of 1.5 billion reads per hour, a significant improvement over the 0.03 billion reads per hour of the default workflow from (5).

**Fig. 2.**
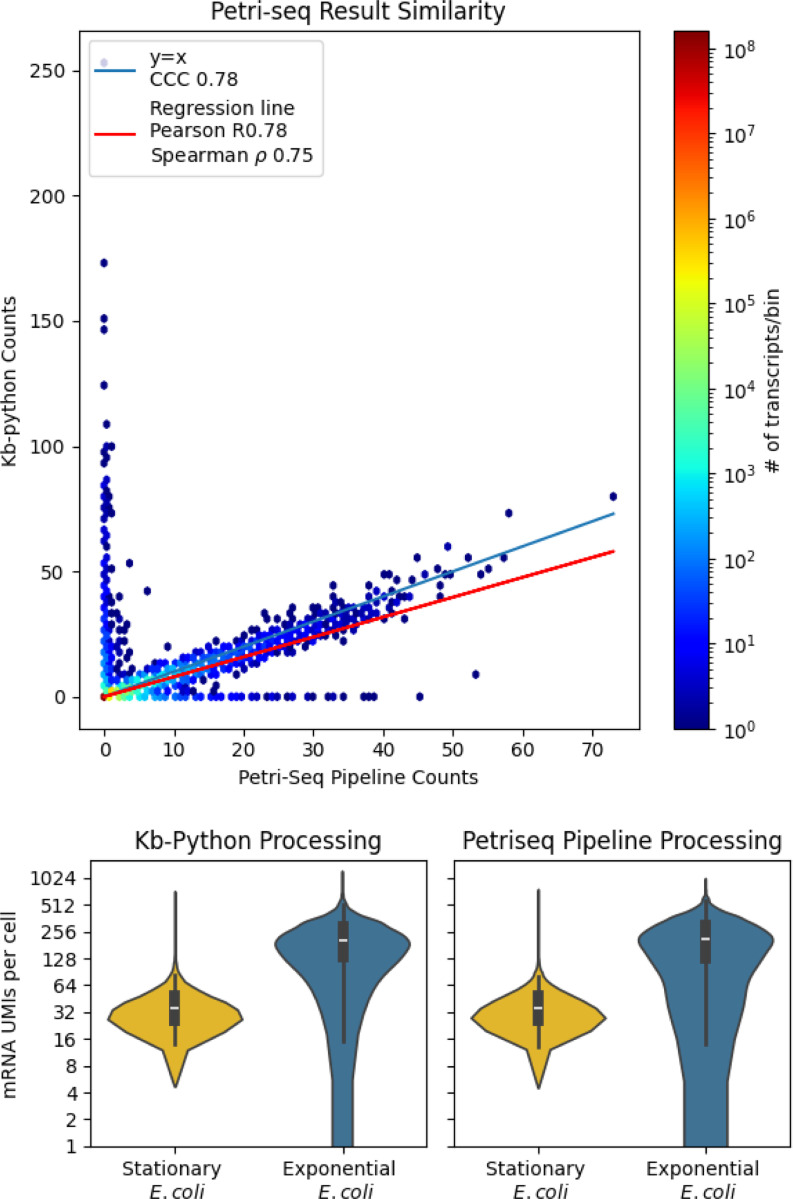
Quantification of the results of using kb-python on PETRI-seq compared to the standard workflow.

After pre-processing the data with kb-python, we found that 54.1 percent of reads pseudoaligned and yielded 16,384 total unique molecular identifiers (UMIs). We were also able to recapitulate the results of Figure 2f from (5) with the count matrices generated by kb-python (Figure 2).

### MATQ-seq.

We next analyzed data from (6). Pre-processing with kb-python proceeded at a rate of 2.3 billion reads per hour, a significant improvement over the 0.1 billion reads per hour of the default workflow of (6).

**Fig. 3.**
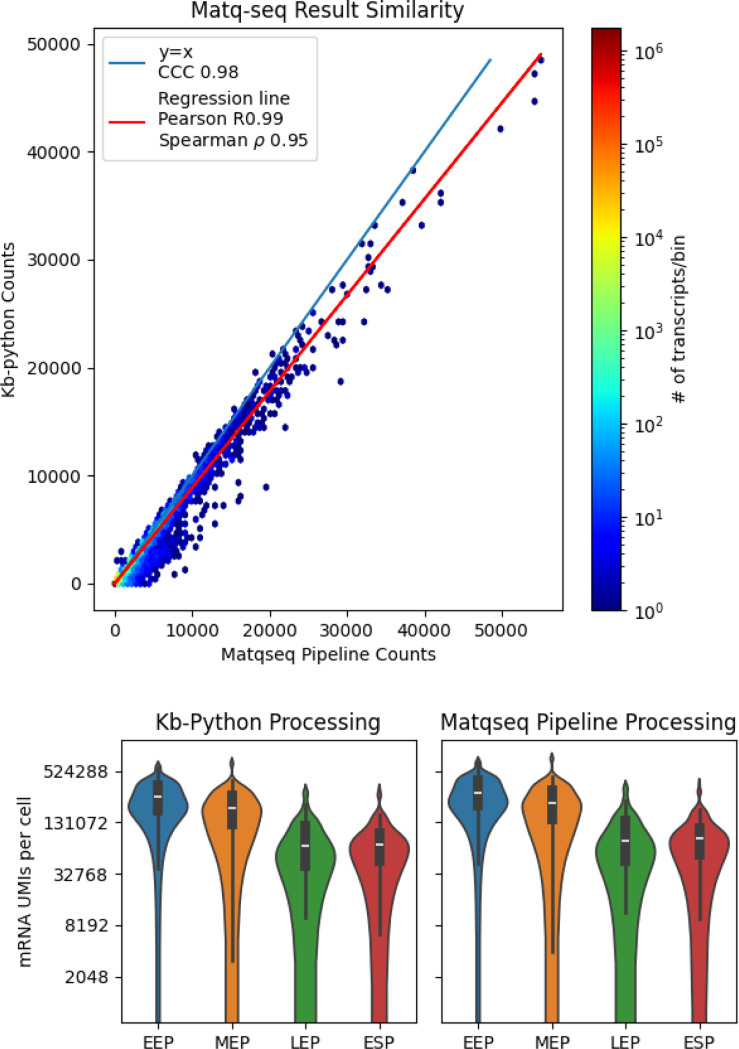
Quantification of the results of using kb-python on MATQ-seq compared to the standard workflow.

After pre-processing with kb-python, we found that 21 percent of reads pseudoaligned, yielding 321,945 UMIs. Our results showed high concordance with those reported using the workflow of (6) (Figure 3).

### BacDrop.

Lastly, we analyzed data from (7). Pre-processing with kb-python proceeded at a rate of 142 million reads per hour, a significant improvement over the 22 million reads per hour of the default workflow of (7).

**Fig. 4.**
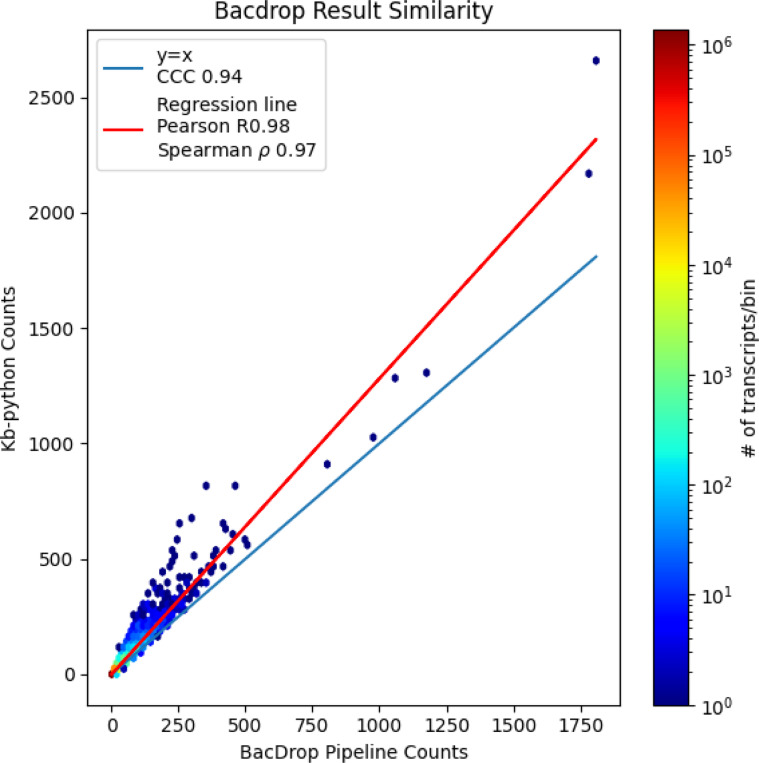
Quantification of the results of using kb-python on BacDrop compared to the standard workflow.

After pre-processing with kb-python, we found that 50.1 percent of reads pseudoaligned, yielding 65,536 UMIs. Our results showed high concordance with those reported using the workflow of (7) (Figure 4).

### k-mer selection and multimapping.

**Fig. 5.**
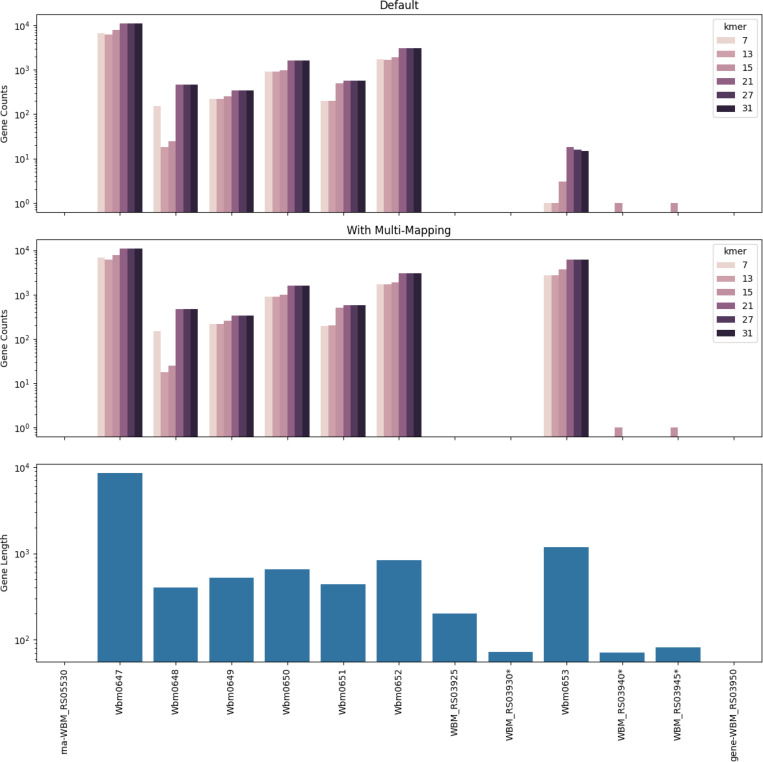
Quantification of the results of using kb-python on bulk RNAseq.

Pseudoalignment has previously been reported to perform poorly on bacterial RNA-seq due to the removal of multi-mapped reads and missing small genes (8). To assess whether that is the case with kb, we assessed performance on a bulk RNAseq dataset from (8). We found that the kallisto built-in multimapping reporting can recover expression that was previously reported as missing (Figure 5). Specifically, we examined the wBm stranded RNA-seq data set and found detection of more reasonable numbers for the gene Wbm0653. However, how best to make use of this feature will be specific to the exact organism/experiment in question, and thus is more a part of the downstream analysis of a potential user.

## Discussion

Our optimized pre-processing workflow demonstrates that kb-python can be effectively adapted for bacterial RNA-seq, making pre-processing highly efficient and accurate. Our kallisto-bustools workflow achieved 100–200X faster pre-processing times than possible with the tools accompanying the single-cell assays, which use traditional alignment-based approaches. Applying kb-python to two different bacterial scRNA-seq datasets, we were able to recapitulate the results from the original methods. Since viral transcripts have similar structure to bacteria, this optimized pre-processing workflow can be used for viral RNA-seq datasets as well.

The advantage of the kallisto-bustools improved processing time cannot be understated, as future biologically relevant datasets are expected to be several orders of magnitude larger (Figure 1). Thus, our tool may help establish workflows that can be robust to the expansion of incoming data in the future (Table 1). Moreover, our approach should be amenable to other single-cell bacterial assays (7)

One limitation of our method that must be considered is its robustness in the presence of contaminants. Single-cell bacterial RNA-seq can include unwanted bacterial organisms in the samples, and while kb-python can make use of indices that include other organisms, performance may be degraded. Unfortunately, the high similarity often seen between bacterial genomes can affect negatively pseudoalignment. This problem will become even more pronounced if the source of the contaminant is unknown.

One advantage of building a bacterial single-cell RNA-seq pre-processing workflow using kb-python is that it facilitates the use of other tools comprising the kallisto-bustools suite. For example, kallisto can be used to identify RdRP encoding viruses (9), making it possibly to perform viral analyses alongside study of bacterial transcriptomics. Future expansion of the viral database could make it more well-suited to investigation of bacteriophages.

## Methods

### Acquisition and preprocessing of PETRI-seq data.

Raw RNA-seq reads from (5) were obtained from GSE141018; specifically GSM4489500/SRR11584024 for PETRI-seq Experiment 2.01. Reference genome Escherichia coli MB165 was used.

Files were passed into the kallisto/bustools workflow. For details and scripts on the workflow, see https://github.com/pachterlab/OBP_2025. The resulting count matrix was then loaded into scanpy for basic filtering.

Reads were also passed into the PETRI-seq workflow following code from https://tavazoielab.c2b2.columbia.edu/PETRI-seq/ using 40000 barcodes. featureCounts_directional_5.py was modified to count on the gene level instead of operon level (feature-Counts -t ‘gene’).

When timing was calculated for the PETRI-seq workflow, genome indexing, quality control with FastQC, and other pre-processing steps, were not included. For kb-python reference index generation was not included.

### Acquisition and preprocessing of MATQ-seq data.

Raw RNA-seq reads from (6) were obtained from https://www.ncbi.nlm.nih.gov/geo/query/acc.cgi?acc=GSE218632. Reference genome https://www.ncbi.nlm.nih.gov/datasets/genome/GCF_000210855.2/ *Salmonella typhimurium* SL13444 was used.

Files were passed into the kallisto/bustools workflow. For details and scripts on the workflow, see https://github.com/pachterlab/OBP_2025. Because MATQ-seq data were provided as individual FASTQ files corresponding to single bactieral cells, substantial computational overhead was incurred from repeated file opening and ckosign operations. To mitigate this inefficiency and to enable more effective use of the kb-python workflow, a random 8bp sequence was appended to the start of each FASTQ file prior to processing. Files where then concatenated into a signle input FASTQ. This 8bp sequence serves as a cell barcode for downstream analysis.

The resulting count matrix was then loaded into scanpy for basic filtering.

The reads were also passed into the MATQ-seq workflow following the code from https://doi.org/10.1038/s41596-025-01157-5.

When timing was calculated for the MATQ-seq workflow, reference indexing was not included. For kb-python reference index generation was not included.

### Acquisition and preprocessing of BacDrop data.

Raw RNA-seq reads from (7) were obtained from GSE141018; specifically GSM5456490/SRR15174657 for the experiment in Fig 4A in (7). Reads were aligned to the https://www.ncbi.nlm.nih.gov/datasets/taxonomy/1328424/ *Klebsiella pneumoniae* BIDMC 35 reference genome.

Following the BacDrop prepreoccesing method, reads were first filtered using UMI-tools to retain only those that contiane both barcode sequences: BC1 (provided in the BacDrop) and BC2 (from the 10X Genomics Chromium Single Cell ATAC barcode whitelist, 737K-cratac-v1.txt.gz, Cell Ranger ATAC v2.2.0). After filtering, UMI, BC1, and BC2 sequences were extracted. Filtered reads were aligned to the reference genome using BWA, gene-level assignments were genetated using featureCounts, and final molecule counts were produced using UMI-tools.

When timing was calculated for the BacDrop workflow, reference indexing was not included. For kb-python reference index generation was not included.

## Figures and Tables

**Table 1.** Benchmarking of the efficiency of kb-python compared to the workflow provided by the creators of each method (including tool version). Benchmarking was performed on an exclusive HPC compute node using scratch space to minimize interference from concerpt jobs. All workflows were run using 10 CPU cores and 50 GB of memory; scripts provided by method creators where modified for this by setting thread and memory threads on genome aligners and counting tools.Time will vary depending on machine, cores, and memory used.

Method and Pipeline	Wall Clocktime (HH:MM:SS)	Reads per minute	Preprocessing/Demultiplexing	Alignment tool	Counting tool	Genome size (nt)	Number of genes in genome	Total reads
PETRI-seq pipeline	03:45:57	~4.5 × 10^5^	Homemade (April 2021)	bwa (v0.7.17)	featureCounts (1.6.3)	~4,641 kb	~4.6 K	1.02 × 10^8^
PETRI-seq kb-python	00:02:00	~5.1 × 10^7^	Kb_python (v0.29.5)	Kb_python (v0.29.5)	Kb_python (v0.29.5)	~4,641 kb	~4.6 K	1.02 × 10^8^
MATQ-seq pipeline	02:48:50	~9.8. × 10^8^	N/A	bowtie2 (v2.5.4)	featureCounts (v2.1.1)	~5,067 kb	~4.8 K	1.65 × 10^9^
MATQ-seq kb-python	00:00:43	~2.3 × 10^9^	Kb_python (v0.29.5)	Kb_python (v0.29.5)	Kb_python (v0.29.5)	~5,067 kb	~4.8 K	1.65 × 10^9^
BacDrop pipeline	04:51:36	~4.4 × 10^6^	umi_tools (2.1.1)	bwa (v0.7.19)	umi_tools (2.1.1)	~5,400 kb	~5.3K	1.29 × 10^9^
BacDrop kb-python	00:14:03	~9.18 × 10^7^	Kb_python (v0.29.5)	Kb_python (v0.29.5)	Kb_python (v0.29.5)	~5,400 kb	~5.3 K	1.29 × 10^9^

## Data Availability

All the code to download data and generate the results of the paper is available at https://github.com/pachterlab/OBP_2025. The repository includes notebooks that can be directly run in Google Colaboratory. The kallisto program is available at https://github.com/pachterlab/kallisto. Documentation is at https://kallisto.readthedocs.io/.
